# Adult hippocampal neurogenesis shapes ideomotor action control in mice

**DOI:** 10.1038/s42003-026-11112-z

**Published:** 2026-10-07

**Authors:** Warsha Barde, Gerd Kempermann, Christian Beste

**Affiliations:** 1 German Center for Neurodegenerative Diseases (DZNE), Dresden, Germany; 2 CRTD – Center for Regenerative Therapies TU Dresden, Dresden, Germany; 3 Cognitive Neurophysiology, Department of Child and Adolescent Psychiatry, Faculty of Medicine, TU Dresden, Dresden, Germany; 4 German Center for Child and Adolescent Health (DZKJ), partner site Leipzig/Dresden, Dresden, Germany

**Keywords:** Cognitive control, Hippocampus

## Abstract

Humans select and learn goal-directed actions through the anticipation of their outcomes. While this ideomotor principle is well established in humans, its biological instantiation and relevance for organizing naturalistic behavior remain unclear, including its neurobiological substrates such as hippocampal plasticity. Here, we leverage high-resolution, continuous behavioral tracking in mice across a month-long series of automated learning tasks to test whether ideomotor principles generalize to naturalistic behavior and how they relate to adult hippocampal neurogenesis. Mice lived in a shared enriched environment and engaged in self-initiated learning tasks, allowing us to quantify how action–outcome associations are formed, maintained, and updated. Despite identical genetic background and environment, animals showed stable individual differences in how these associations were organized. These differences correlated with levels of adult hippocampal neurogenesis: higher neurogenesis was associated with broader, more variable exploration and greater flexibility in updating action–outcome relations, whereas lower neurogenesis was linked to more rigid behavior. Together, these findings link ideomotor theory to a cellular plasticity mechanism supporting flexible action control.

## Abstract (web summary)

Longitudinal IntelliCage monitoring shows how mice form, retrieve and update action–outcome associations during self-initiated behavior. Stable individual differences in exploratory organization and behavioral flexibility covary with adult hippocampal neurogenesis, linking cellular plasticity to ideomotor action control.

## Introduction

Genetically identical (inbred) mice, even when housed in an identical environment, develop markedly individualized behavioral patterns^1^, including trajectories of exploratory behavior, learning patterns, and social interactions. The individual engagement of each mouse with the environment, including its social and physical aspects, appears to play a critical role in shaping these patterns.

Basic forms of behavior, such as those expressed by these mice, may provide a particularly direct, yet still to be tested, window into general principles of action. Ideomotor theory^2–4^ proposes that actions are selected through the anticipation of their effects^5–8^ and that these anticipations are learned through self-generated interaction with the environment^9^. Once acquired, action-effect couplings can be reactivated by internal representations that specify the currently desired action-effect (i.e., goals^10^). These action-effect representations are referred to as event files^6,11^. Critically, event files are not static memory traces but are dynamically managed through binding and retrieval processes for which interrelated neurophysiological processes have been described^12–15^. Binding stands for the integration of action-related, sensory, contextual, and internal-state features into a coherent event file during experience, whereas retrieval reactivates these bindings when overlapping features or anticipated effects are encountered again^16^. Through repeated cycles of binding and retrieval, action-effect representations become selectively strengthened, weakened, or updated, thereby shaping the repertoire of actions that are most readily selectable in a given context^12,16,17^. From this perspective, the exploratory engagement of mice can be understood as an ideomotor learning process, in which self-initiated interactions continuously generate new opportunities for binding and retrieval, progressively structuring and individualizing event-file repertoires. Accordingly, the individualized behavioral trajectories observed in genetically identical mice, in which genetic sources of variance in behavior are controlled for^1^, can be conceptualized as the gradual differentiation of event-files under identical external conditions (the so-called “shared environment”). Each mouse’s engagement with its environment determines which action-effect contingencies are bound together, how often they are retrieved, and how flexibly they can be recombined when contingencies change. Over time, this leads to increasing systematic inter-individual differences in behavior, because of, as we hypothesize, differences in the structure, stability, and accessibility of learned action-effect couplings stored in event files. In this way, basic exploratory, social, and learning behaviors in mice may instantiate the same organizing principles of action that ideomotor theory has shown for humans. Yet, whether and how such binding- and retrieval-based ideomotor mechanisms shape the emergence of stable, individualized behavioral trajectories in lower-level vertebrates has as yet remained an open question.

**Fig. 1:**
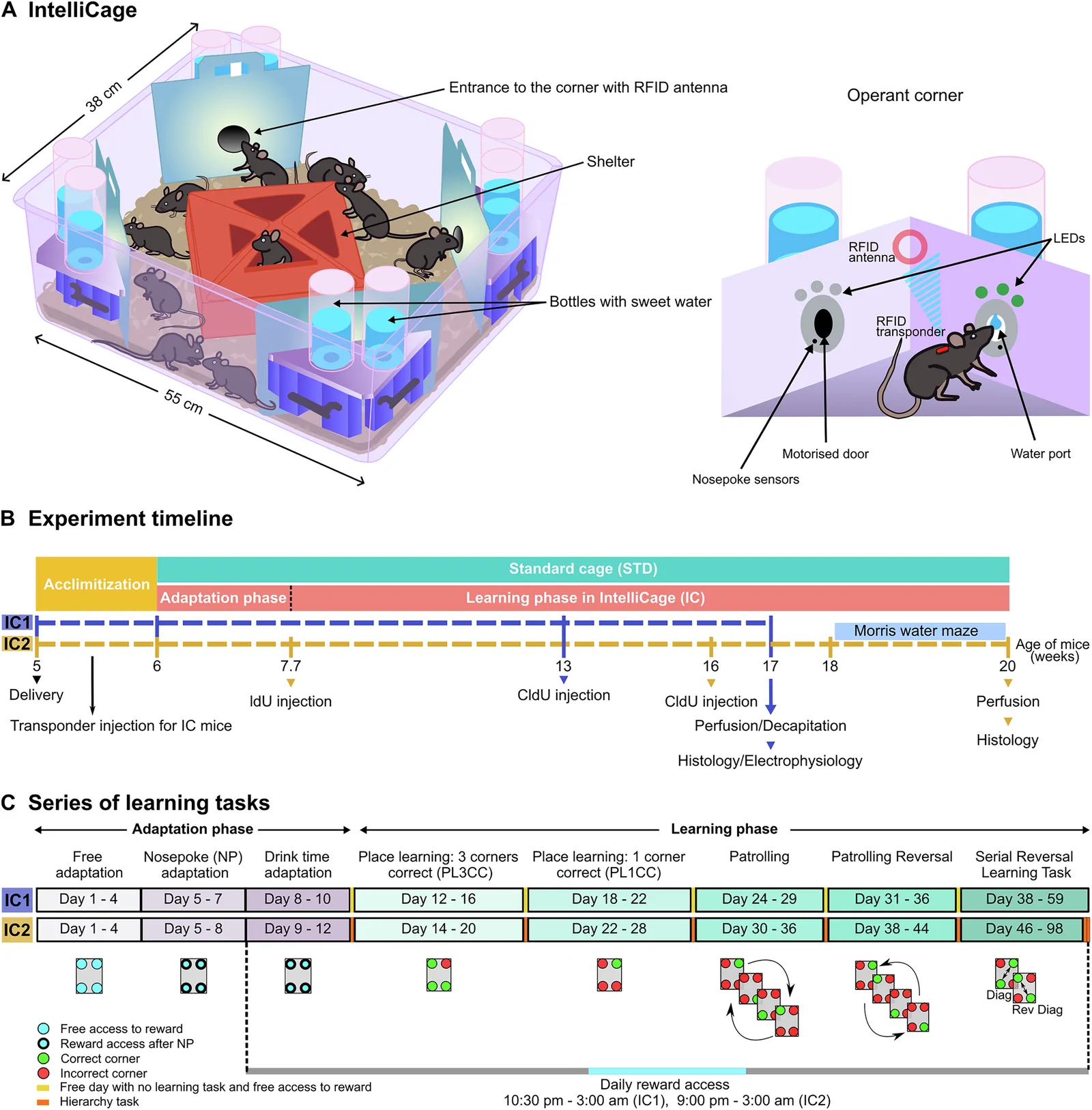
IntelliCage and experimental timeline. **A** Schematic overview of the IntelliCage (IC) that accommodates up to 16 mice at a time and is equipped with four operant conditioning corners with RFID antennas for animal identification (left panel). Each corner consists of two nosepoke ports with motorized doors, LEDs above the doors, and access to sweetened water as a reward behind the door (right panel). **B** Ninety-six female C57BL/6JRj mice (5 weeks old), across two experiments, were randomly assigned to IC (IC1: *n* = 16; IC2: *n* = 32) or standard housing (STD1: *n* = 16; STD2: *n* = 32) for 11–14 weeks. In IC2, two IC setups were used simultaneously, and 8 of the 32 mice were learning-deprived (excluded from the current analyses). Adult hippocampal neurogenesis was quantified using thymidine analog labeling: in IC1, all mice received 5-chlorodeoxyuridine (CldU) injections 4 weeks before the end of the experiment; in IC2, mice received 5-Iododeoxyuridine (IdU) and CldU injections 12.7 and 4 weeks before the end of the experiment, respectively. During the final two weeks, all mice were additionally tested in the reference-memory version of the Morris water maze, however, these data fall outside the scope of the present study. **C** The experimental protocol consisted of an initial adaptation phase, designed to familiarize mice with the apparatus and reward contingencies, including free adaptation, nose-poke adaptation, and drinking-time adaptation. This was followed by a learning phase comprising five sequential place-learning tasks. Learning tasks were interspersed with free days in IC1, and a competitive social dominance task to assess social hierarchy in IC2, however, the latter addressed a separate research question and falls outside the scope of the current study.

If ideomotor principles are realized through binding and retrieval processes, their defining signatures should emerge gradually across extended sequences of self-generated behavior. Such processes unfold over timescales that align with experience-dependent neural plasticity^18–20^, particularly within the hippocampus, where structural remodeling and adult neurogenesis have been linked to exploratory engagement and learning flexibility^21,22^. Tracing these trajectories of action-effect learning and their neural substrates at this fine-grained level is not feasible in humans. As a result, much of ideomotor theorizing has necessarily relied on short-term, trial-based paradigms and indirect neural measures. Animal models, therefore, provide an essential opportunity to examine how ideomotor principles are built, maintained, and reorganized over time, and to link behavioral trajectories directly to plasticity mechanisms that remain inaccessible in human research. Longitudinal, ecologically valid behavioral data are required to study how ideomotor principles unfold through self-generated interaction with the environment. To this end, we used the IntelliCage (IC)^23^, a fully automated home-cage system that enables continuous assessment of exploratory, learning, and social behavior in group-housed mice under minimally invasive conditions (Fig. 1A). The IC consists of four operant corners equipped with nose-poke ports and automated doors that control access to sweetened water rewards. All visits, nose pokes, and licks are automatically logged via RFID-based identification, enabling the capture of self-initiated decisions over extended time periods without experimenter intervention. Across two experiments, 96 five-week-old female C57BL/6JRj mice were randomly assigned to IC (IC1^1,24^: *n* = 16; IC2: *n* = 32) or standard housing (STD1: *n* = 16; STD2: *n* = 32) and monitored for 11–14 weeks (Fig. 1B). In IC2, 8 of 32 mice were assigned to a learning-deprived condition resulting in a total of 40 learning mice from both the IC experiments that were included in the current analyses. Following an initial adaptation phase, mice completed a sequence of place learning and reversal tasks with changing reward contingencies (Fig. 1C), spanning stable, shifting, and alternating action-effect mappings. This design yields high-resolution behavioral time series across exploration, acquisition, adaptation, and change of contingency and provides a naturalistic framework to examine how goal-directed behavior, learning flexibility, and individuality emerge over time.

If ideomotor principles are instantiated through binding and retrieval processes^12,16,17^, then experience-dependent neural plasticity should relate selectively to behavioral markers that index these processes. Binding-related aspects of action-effect learning capture how diverse features of an action episode are integrated into coherent event files. Retrieval-related aspects reflect how previously formed event files are reactivated to guide future behavior under changing demands. These two components place different demands on representational plasticity: while binding requires the formation and differentiation of new action–effect associations, retrieval emphasizes the flexible reuse and updating of existing ones. Adult hippocampal neurogenesis is as a process of structural network plasticity well-positioned to contribute to both aspects. New granule cells have been implicated in experience-dependent representational differentiation^21,25,26^ and in maintaining flexibility in the face of interference^27,28^. These functions align with the continuous formation, refinement, and reconfiguration of event files as suggested by human neurophysiological studies^13,29–32^. Accordingly, adult hippocampal neurogenesis should covary with behavioral parameters that reflect how richly event file repertoires are bound and how flexibly they are retrieved over time.

## Results

### Strength of feature binding scales with exploratory breadth

Exploration reflects traits such as curiosity or novelty-seeking. Exploration plays an integral role in shaping both the formation and accessibility of event-files^16,17^. Specifically, the breadth and variability of exploratory behavior could determine how many stimulus-response-effect (S-R-E) combinations are experienced, encoded, and subsequently retrieved. Within the IntelliCage, each visit constitutes a multi-feature action episode (location, action and outcome). Binding depends on how often and how diversely these features co-occur. High exploration reflects broad sampling of the available action–effect space, leading to exposure to a larger number of distinct S–R–E combinations. In contrast, low exploration reflects repeated sampling of a restricted subset of states, increasing the likelihood that specific feature combinations are repeatedly co-activated and therefore more strongly bound.

**Fig. 2:**
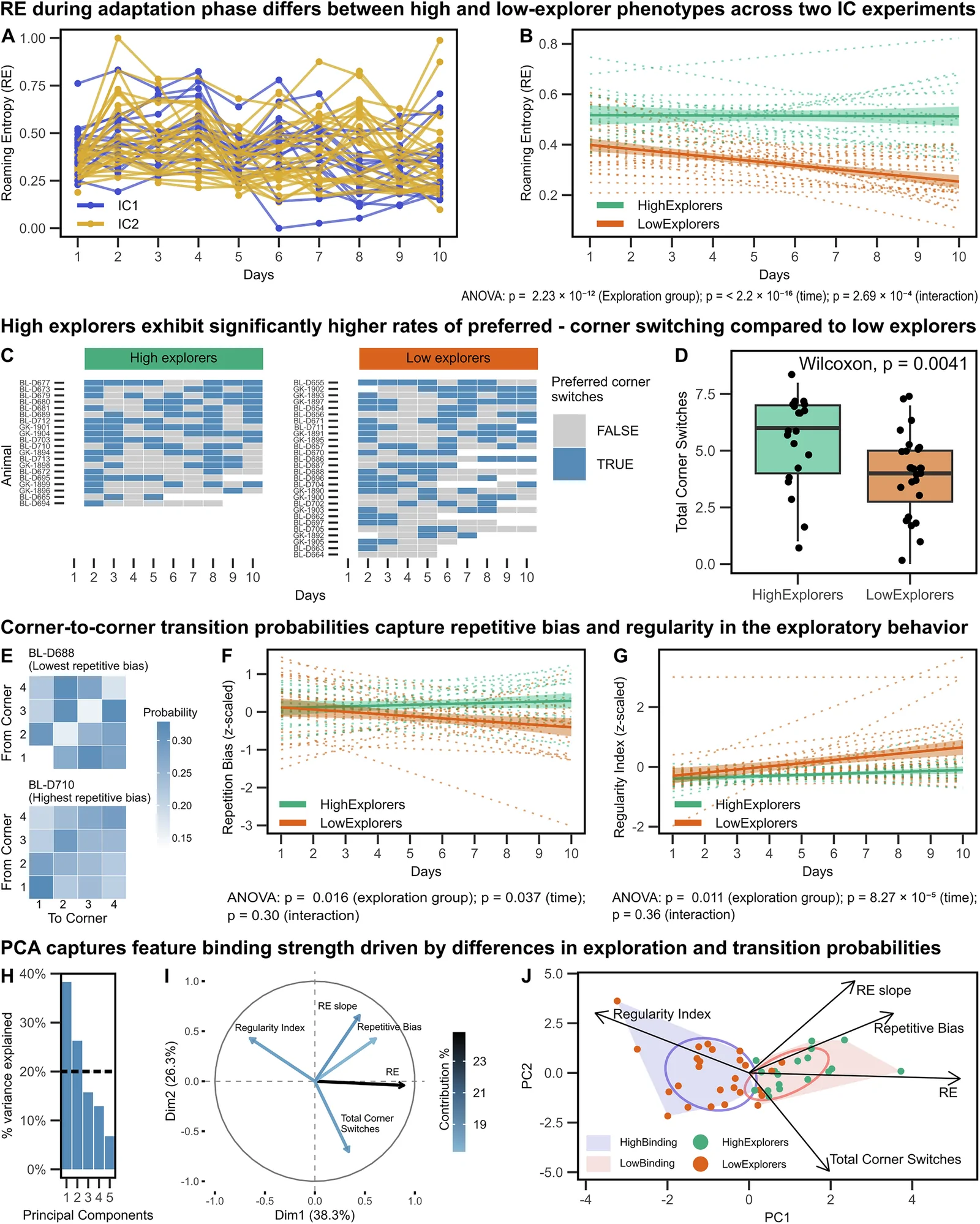
Feature binding strength scales with exploratory breadth. **A** Individual trajectories of roaming entropy (RE) over 10 adaptation days from two IC experiments. **B** RE across days with linear fits, showing sustained high exploration in high explorers and declining exploration in low explorers. Solid lines represent group mean; shaded bands represent the s.e.m. A linear mixed-effects model was used with exploration group, day and their interaction as fixed effects and animal as a random effect. Two-sided Type III F-tests with Satterthwaite’s approximation showed significant effects of exploration group (F(1, 45.91) = 89.80, *P* = 2.23 × 10⁻¹²), day (F(9, 412.96) = 12.84, *P* = 4.46 × 10⁻¹⁸) and their interaction (F(9, 412.96) = 3.58, *P* = 2.69 × 10⁻⁴). **C, D** Preferred corner switches across consecutive days, showing higher switching frequency in high explorers (two-sided Wilcoxon rank-sum test, *P* = 0.0041; n = 40; IC1 = 16; IC2 = 24). Box plots show the median (centre line), interquartile range (box) and values within 1.5 × the interquartile range (whiskers). Individual data points are shown as dots. **E** Corner-to-corner transition matrices capture additional exploratory patterns like repetitive bias and regularity index. Examples of transition matrices for two animals with lowest and highest repetitive bias. **F** High explorers exhibit increased repetition bias, re-visiting the same corner while maintaining uniform visit distribution across corners. Solid lines represent group mean. and shaded bands represent s.e.m. A linear mixed-effects model was used with exploration group, night and their interaction as fixed effects and animal as a random effect. Two-sided Type III F-tests with Satterthwaite’s approximation showed significant effects of exploration group (F(1, 45.83) = 6.23, *P* = 0.016) and night (F(9, 412.91) = 2.01, *P* = 0.037), but no exploration group × night interaction (F(9, 412.91) = 1.19, *P* = 0.30). **G** Low explorers exhibit high regularity index indicating more visit stereotypy and structured sequences. Solid lines represent group mean. and shaded bands represent s.e.m. A linear mixed-effects model with exploration group, night and their interaction as fixed effects and animal as a random effect. Two-sided Type III F-tests with Satterthwaite’s approximation showed significant effects of exploration group (F(1, 44.90) = 7.06, *P* = 0.011) and night (F(9, 411.92) = 3.93, *P* = 8.27 × 10⁻⁵), but no exploration group × night interaction (F(9, 411.92) = 1.10, *P* = 0.36). **H** Scree plot of PCA showing the percentage of variance explained by each principal component. **I** PCA loadings for five behavioral metrics: RE, RE slope, corner switching, repetition bias, and regularity index. **J** Median split of PC1 scores classifies high- and low-feature-binding animals; convex hulls and density contours indicate partial overlap with high- and low-explorer categories.

During the adaptation phase, when all corners were equally accessible and rewarded, with no spatial bias or task constraints, roaming entropy served as a measure of the uniformity and diversity of spatial exploration across individuals (Fig. 2A)^33,34^. Roaming entropy (RE) is the probability that a mouse is found at a given location at a given time, thereby linking physical exploratory activity to territorial coverage. Based on the slope and mean of RE, animals were classified as high explorers and low explorers (Fig. 2B). To examine how exploratory behavior evolved across the adaptation phase, we fitted a linear mixed-effects model with exploration group (high vs. low explorers) and day as fixed effects, and animal identity as a random effect to account for repeated measurements. We observed a strong main effect of exploration group (F(1, 45.91) = 89.80, *p* = 2.23 × 10⁻¹²), indicating that high and low explorers differed in their overall levels of RE. There was also a significant main effect of day (F(9, 412.96) = 12.84, *p* < 2.2 × 10⁻¹⁶), demonstrating that exploratory behavior changed systematically across the adaptation period. We observed a significant exploration group × day interaction (F(9, 412.96) = 3.58, *p* = 2.69 × 10⁻⁴), indicating that the temporal dynamics of exploration differed between high and low explorers. High explorers consistently showed higher RE values throughout the adaptation period, reflecting a broader and more evenly distributed sampling of all available corners. Suggesting that these animals are likely forming a wider range of stimulus–response associations in the absence of specific task demands. In contrast, low explorers showed a decline in RE over days, indicating a progressive restriction of their exploratory range. Rather than sampling the environment uniformly, these animals likely limited their activity to a subset of corners, reflecting the early appearance of spatial biases or habitual preferences. These differences likely set the stage for the behavioral flexibility differences observed in later task phases. Mice also differed in the stability of spatial preferences. While all animals formed preferred “home-base” corners, high explorers switched their preferred corners significantly more often than low explorers (Fig. 2C, D; two-sided Wilcoxon rank-sum test, *p* = 0.0041), consistent with weaker bindings between location and action.

To further characterize the structure underlying the exploratory behavior, probability matrices for corner-to-corner transitions were constructed for each subject, capturing the frequency with which a given corner was visited after leaving another. Diagonal entries reflect the tendency to re-enter the same corner, providing a measure of repetitive bias (Fig. 2E). Off-diagonal entries quantify transitions between different corners. From the off-diagonal entries, a regularity index was computed as the mean sum of squared residuals between observed and expected transition frequencies across all non-same corner transitions. Higher values indicated more stereotyped and predictable visiting sequences^35^. High and low explorers differed in both repetitive bias and behavioral regularity (Fig. 2F, G). A linear mixed-effects model on repetition bias revealed a significant main effect of exploration group (*F*(1, 45.83) = 6.23, *p* = 0.016), with high explorers showing greater repetition bias than low explorers. There was also a modest effect of night (*F*(9, 412.91) = 2.01, *p* = 0.037), indicating some variation across the adaptation period, but no exploration group × night interaction (*F*(9, 412.91) = 1.19, *p* = 0.30). Analysis of the regularity index similarly revealed a significant main effect of exploration group (*F*(1, 44.90) = 7.06, *p* = 0.011), with low explorers exhibiting higher regularity than high explorers. In addition, there was a strong main effect of night (*F*(9, 411.92) = 3.93, *p* = 8.27 × 10⁻⁵), indicating that the structure of visit sequences evolved over time, but no interaction between exploration group and night (*F*(9, 411.92) = 1.10, *p* = 0.36). Together, these results indicate that high explorers tend to revisit the same corner while also uniformly distributing their visits across corners, whereas low explorers exhibit more stereotyped and structured corner visit patterns.

To reduce the dimensionality of the behavioral metrics and derive a composite score that would reflect each animal’s feature binding strength we performed a principal component analysis (PCA) on five behavioral summary measures—RE, slope of RE, preferred corner switching frequency, repetition bias, and regularity index. The first two principal components captured the largest proportion of variance in the dataset, as shown by the scree plot (Fig. 2H). PC1 was characterized by strong positive contributions from RE (0.65), repetitive bias (0.44), RE slope (0.32) and preferred corner switching frequency (0.25), coupled with a strong negative loading from regularity index (−0.47) (Fig. 2I and Table S1). High PC1 scores thus reflect animals with high RE, repetition tendencies, steeper temporal changes in RE, and lower behavioral regularity. In this way, PC1 best represents binding strength as it captures a behavioral gradient ranging from flexible, high-entropy, and variable behavior to stereotypical, predictable, low-entropy behavior. Animals were classified into high- and low-feature-binding groups using the median PC1 score as a threshold, yielding two distributions in PCA space, as visualized by density contours and convex hulls (Fig. 2J). Although PCA-based clustering did not produce fully discrete groups, the distribution along PC1 overlapped substantially with prior high- and low-explorer classifications. High and low explorers differed significantly on PC1 (Welch t-test: *t* (38.43) = 6.99, *p* < 2.419 × 10^−8^), with high explorers having higher PC1 scores (mean = 1.20) than low explorers (mean = −0.83). This indicates that the composite feature-binding score captures meaningful variance consistent with exploration tendencies, reflecting a gradient from broad, variable, and less stereotyped exploration to restricted, repetitive, and structured behavior.

This dimension does not merely reflect how much animals explore, but how their experience is structured over time. The formation of event files depends on how often and how diversely A feature combinations co-occur. Animals with high RE and frequent switching sample a wide range of action–environment combinations, limiting repeated co-activation of the same features and thus preventing the dominance of a small set of strongly bound event files. In contrast, animals with low RE and high regularity repeatedly experience the same action–context combinations, promoting the stabilization of more rigid and strongly bound associations.

Accordingly, we interpret PC1 as an axis of exploratory organization that constrains the diversity and repetition structure of action–effect experiences, which in turn shapes the formation of event files. These measures are not independent behavioral readouts but form structured relationships consistent with theoretical expectations of binding and retrieval processes.

### Strength of feature retrieval scales with flexible learning

The learning tasks increased progressively in difficulty. Mice initially learned the location of rewarded corners, and in subsequent tasks were required to unlearn prior corner assignments to acquire newly rewarded locations. Both learning and persistence are informative about retrieval processes. Continued visits to previously rewarded corners, a measure of persistence, captures the continued influence of prior action–effect associations. In contrast, successful performance in the current task indicates the acquisition of updated contingencies.

**Fig. 3:**
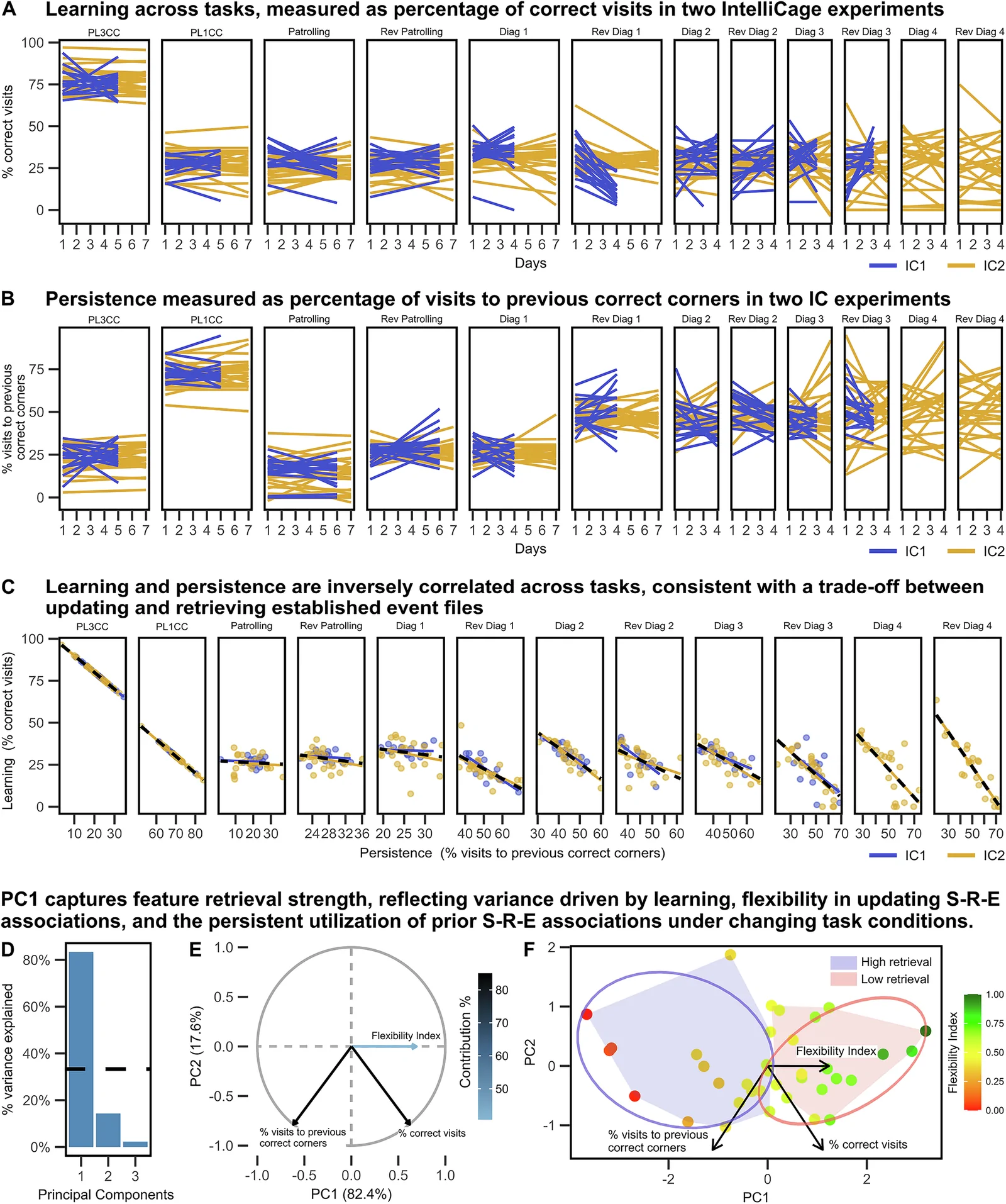
Feature retrieval strength scales with flexible learning. **A** Individual learning trajectories over successive tasks quantified as percentage of correct visits showing both fanning out and fanning in of trajectories reflecting inter-individual differences in learning performance that are shaped by task contingencies. **B** Persistence behavior, quantified as the percentage of visits to previously rewarded corners during new tasks, showing both fanning-out and fanning-in patterns across tasks. **C** Negative correlation between learning success (% correct visits) and persistence (% visits to previous correct corners), illustrating the trade-off between updating and retrieving previously established event files. **D** Scree plot from PCA of three behavioral metrics: % correct visits, % visits to previous correct corners and flexibility index, showing variance explained by each component. **E** PCA loadings, with PC1 positively weighted for flexibility index and % correct visits and negatively weighted for % visits to previously correct corners. **F** Classification of animals into high- and low-feature-retrieval groups using the median PC1 score. Density contours and convex hulls visualize the separation, showing variance in flexibility index and inter-individual differences in feature retrieval strength. High PC1 scores reflect animals that flexibly update behavior, retrieve relevant prior information, and minimize interference from obsolete event files.

Individual learning success was quantified as the percentage of correct visits. Across the entire learning phase, trajectories diverged and variance increased, reflecting inter-individual differences in learning (Fig. S1). Within single tasks, learning curves showed both fanning-out and fanning-in patterns, consistent with our previous reports^1^ (Fig. 3A). To look at the persistence behavior of mice, we calculated the percentage of times mice visited the previously correct corner during a new task. The persistence curves of individual mice also showed a fanning-out or fanning-in pattern depending on the task (Fig. 3B). Learning and persistence measures were negatively correlated across tasks, consistent with a trade-off between updating and retrieving established event files (refer to S2 Table for statistical details). Mice that frequently revisited previously correct corners exhibited lower learning success, and vice versa (Fig. 3C). To quantify this trade-off, a flexibility index per animal was calculated as the difference between learning score and persistence score across tasks (Fig. S2), which showed that animals exhibited stable inter-individual differences in their flexible learning.

To derive a composite measure of feature retrieval strength, PCA was performed on three behavioral metrics: flexibility index, percent correct visits, and percent visits to previous correct corners. The first two principal components captured the largest proportion of variance in the dataset, as shown by the scree plot (Fig. 3D). PC1 explained positive contributions from flexibility index (0.62) and percent correct visits (0.56) and negative contributions from percent visits to previous correct corners (−0.56) (Fig. 3E and Table S3). High PC1 scores, therefore, reflected animals that flexibly updated their behavior, successfully retrieved relevant prior information, and minimized retrieval of now-irrelevant past event files. Animals were classified into high- and low-feature-retrieval groups using the median PC1 score as a threshold, resulting in two distributions in PCA space, visualized as density contours and convex hulls (Fig. 3F). The distribution along PC1 captured meaningful variance in flexibility index. PC1 scores were strongly positively correlated with behavioral measures of flexibility (*r* = 0.93, 95% CI [0.86, 0.96], *p* = 3.20 × 10^−16^) and performance (*r* = 0.89, 95% CI [0.80, 0.94], *p* = 3.19 × 10^−13^). Conversely, PC1 was strongly negatively correlated with the persistence score (r = −0.89, 95% CI [−0.94,−0.80], *p* = 2.63 × 10^−13^), indicating that variance in PC1 scores reflects variance in feature retrieval strength (Fig. S3). PC1 represents a continuous axis along which animals differ in their tendency to retrieve previously learned action–effect associations versus updating behavior in response to new contingencies.

### Adult hippocampal neurogenesis correlates positively with weak feature binding and retrieval strength

Adult hippocampal neurogenesis contributes to the flexible integration of new information into pre-existing contexts by supporting behavioral pattern separation and the avoidance of catastrophic interference. We hypothesize that the improved updating would also support the formation and adjustment of event files. Environmental enrichment not only increases the mean levels of adult hippocampal neurogenesis in mice but also amplifies inter-individual variability compared to standard housing, a pattern correlated with the development of stable, individualized behavioral trajectories, as shown in previous experiments^1,34,36^. To quantify individual rates of adult neurogenesis, all mice received injections of a thymidine analog, allowing subsequent identification of newly generated cells via immunohistochemistry. In the IC1 cohort, all mice received CldU injections 4 weeks before the end of the experiment (late phase); in the IC2 cohort, mice received IdU and CldU injections, 12.7 (early phase) and 4 weeks (late phase) before the end of the experiment, respectively. Thymidine analog labeling identifies cells that underwent DNA synthesis during the respective labeling period and survived until tissue collection. Because co-labeling with neuronal markers was not performed in the present study, the histological measure is interpreted as adult newborn hippocampal cells rather than as a count of mature adult-born neurons. Quantification of IdU- and CldU-positive cells in the dentate gyrus (DG) at both the early phase and the late phase revealed that IC mice had significantly higher neurogenesis than STD mice (Fig. S4). There was an expected age-related decrease in adult hippocampal neurogenesis between early and late phase, as well as an interaction between time and housing condition (time: *F*(1,119) = 560.33, *p* = 7.77 × 10^−47^, ges = 0.825; interaction: *F*(1,119) = 4.013, *p* = 4.7 × 10^−2^, ges = 0.033)^34^.

**Fig. 4:**
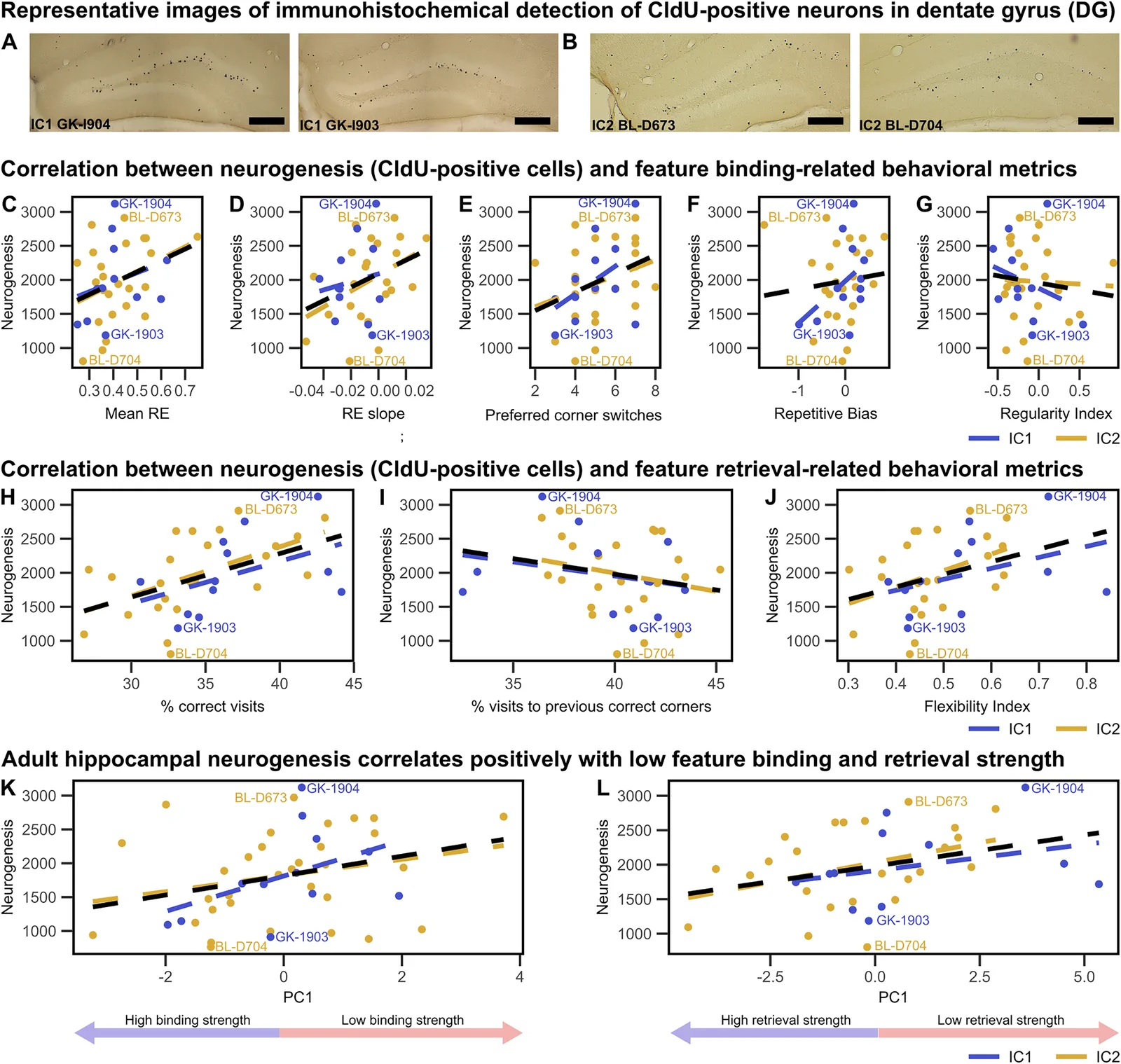
Adult hippocampal neurogenesis correlates positively with weak feature binding and retrieval strength. **A** Representative dentate gyrus (DG) sections showing CldU-positive cells from mice with the highest and lowest levels of neurogenesis in the IC1 cohort. Scale bars, 100 µm. **B** Representative DG sections showing CldU-positive cells from mice with the highest and lowest levels of neurogenesis in the IC1 cohort. Scale bars, 100 µm. **C** Mean RE showed a positive but non-significant association with hippocampal neurogenesis after FDR correction (*r* = 0.32, *p* = 0.054, adjusted *p* = 0.090). **D** RE slope showed a nominally significant positive correlation with hippocampal neurogenesis that was non-significant after FDR correction (*r* = 0.34, *p* = 0.041, adjusted *p* = 0.090). **E** Preferred corner switching frequency showed a significant positive correlation with hippocampal neurogenesis that did not remain significant after FDR correction (*r* = 0.36, *p* = 0.033, adjusted *p* = 0.090). **F** Repetition bias showed a non-significant positive correlation with hippocampal neurogenesis (*r* = 0.11, *p* = 0.54, adjusted *p* = 0.537). **G** Regularity index showed a non-significant negative correlation with hippocampal neurogenesis (*r* = −0.12, *p* = 0.50, adjusted *p* = 0.537). **H** Learning score was significantly positively correlated with hippocampal neurogenesis after FDR correction (*r* = 0.50, *p* = 0.002, adjusted *p* = 0.00612). **I** Persistence showed a negative but non-significant correlation with hippocampal neurogenesis (*r* = −0.23, *p* = 0.18, adjusted *p* = 0.182). **J** Flexibility index was significantly positively correlated with hippocampal neurogenesis after FDR correction (*r* = 0.38, *p* = 0.024, adjusted *p* = 0.0358). **K** Low feature binding strength correlates positively with hippocampal neurogenesis (*r* = 0.30, *p* = 0.047). **L** Low feature retrieval strength correlates positively with hippocampal neurogenesis (*r* = 0.34, *p* = 0.044). For detailed statistics of correlation analysis refer to Table S4. For rank of animals according to behavioral metrics refer to Tables S5 and S6.

Correlational analyses demonstrated a clear relationship between the number of newborn neurons in the dentate gyrus and behavioral measures reflecting feature binding and retrieval. This analysis used the CldU-positive cells because the timepoint of CldU injection at 4 weeks before the end of the experiment yields information about a cohort of new neurons in a network situation after the learning experience. Neurogenesis was positively correlated with indicators of feature binding strength that capture exploratory breadth and variability - like RE (Fig. 4C; *r* = 0.32, *p* = 0.054, adjusted *p* = 0.090), the slope of RE over time (Fig. 4D; *r* = 0.34, *p* = 0.041, adjusted *p* = 0.090), frequency of preferred-corner switching (Fig. 4E; *r* = 0.36, *p* = 0.033, adjusted *p* = 0.090). These nominally significant positive correlations, however, didn’t remain statistically significant after FDR correction. Hippocampal neurogenesis showed a non-significant positive correlation with repetition bias (Fig. 4F; *r* = 0.11, *p* = 0.54, adjusted *p* = 0.537) and negative correlation with regularity index (Fig. 4G; *r* = −0.12, *p* = 0.5, adjusted *p* = 0.537). This shows that higher levels of adult-born neurons are associated with less rigid and more variable exploration patterns. Together, these correlations indicate that enhanced adult neurogenesis supports weaker feature binding, which was further supported by the positive correlation with the composite feature-binding score derived from the first principal component (Fig. 4K; *r* = 0.34, *p* = 0.044). Adjusted *p*-values were computed using false discovery rate (FDR) correction across all behavioral correlations to account for multiple comparisons.

Similarly, adult hippocampal neurogenesis was positively associated with metrics reflecting feature retrieval strength. Mice with more newborn neurons demonstrated significantly better learning performance (Fig. 4H; *r* = 0.5, *p* = 0.002, adjusted *p* = 0.00612) and a higher flexibility index (Fig. 4J; *r* = 0.38, *p* = 0.024, adjusted *p* = 0.0358), reflecting an enhanced ability to adjust behavior in response to changing task demands. At the same time, persistence showed a negative, but statistically non-significant, relationship with neurogenesis (Fig. 4I; *r* = −0.23, *p* = 0.18, adjusted *p* = 0.182), a tendency to maintain previously learned responses despite changing contingencies. This pattern was also evident in the composite retrieval score derived from PCA, which indicated that higher neurogenesis was associated with weaker feature retrieval strength (Fig. 4L; *r* = 0.30, *p* = 0.047). Collectively, these findings suggest that higher levels of adult hippocampal neurogenesis are associated with more adaptive updating of event-files and less rigid retention of previously encoded associations.

## Discussion

The present work addresses whether core principles of ideomotor theory, developed for humans, operate as organizing mechanisms also of murine behavior in a semi-naturalistic experimental model and whether these mechanisms are instantiated in experience-dependent hippocampal plasticity that might structurally underlie the emergence of stable behavioral individuality. By translating ideomotor concepts into longitudinal measures of self-generated behavior in mice and relating them to adult hippocampal neurogenesis, we show that binding- and retrieval-related parameters capture systematic structure in exploration and learning trajectories. This establishes a direct link between ideomotor principles and cellular plasticity mechanisms. From this perspective, ideomotor principles provide a mechanistic framework for how individualized behavioral trajectories emerge, while hippocampal neurogenesis offers a biological substrate through which these principles are instated.

Within an ideomotor framework, exploration constitutes the primary learning process through which action-effect representations are acquired and structured^11,37–39^. Self-generated exploratory behavior provides repeated opportunities to sample, co-activate, and bind relations between actions, spatial contexts, and outcomes into event files. Consistent with this view, early exploration during the adaptation phase predicted later differences in behavioral organization, indicating that exploration lays the groundwork for the formation and stabilization of event file repertoires. Measures such as roaming entropy, corner switching, and transition structure therefore index how broadly and flexibly animals sample the available action-effect space. Importantly, variation in exploration translated into systematic differences in binding-related behavior. Animals exhibiting broad, variable exploration developed weaker, more flexible bindings between actions and locations, whereas animals with restricted, stereotyped exploration formed stronger, more rigid bindings. From an ideomotor perspective, extensive exploration increases the diversity of binding episodes, limiting the dominance of a small set of event files and preserving flexibility, whereas restricted exploration promotes repeated co-occurrence of the same features, strengthening specific bindings and constraining behavioral variability. In this way, exploration shapes the representational landscape, within which later learning and action selection can operate. At the same time, our findings corroborate the core prediction that binding and retrieval constitute separable, though interacting, dimensions of action control^17^. Exploratory behavior primarily indexed binding-related processes, whereas learning under changing contingencies primarily indexed retrieval-related processes. The partial independence of these dimensions shows that differences in how action-effect relations are bound during exploration do not trivially determine how strongly these relations are later retrieved during learning. These findings suggest that behavioral individuality reflects differences in how action-effect representations are structured and accessed over time. Even under identical genetic and environmental conditions, animals develop distinct event-file repertoires because self-generated experience systematically biases which action-effect relations are bound, how strongly they are stabilized, and how flexibly they can later be retrieved. Individuality thus likely emerges from ideomotor learning. Individualized behavioral trajectories arise from differences in the organization and reuse of event files, determining action selection.

A central contribution of the present work is the link of ideomotor principles to experience-dependent hippocampal plasticity. Adult hippocampal neurogenesis has been repeatedly associated with exploratory engagement^18,19,36^, representational differentiation^22,26^, and learning flexibility^21^, yet its role in shaping action-control organization has not yet been specified. Our findings suggest that adult neurogenesis specifically covaries with ideomotor-relevant features of behavior (i.e., the strength of binding and retrieval of action-effect representations/event files). Higher levels of adult hippocampal neurogenesis were associated with broader and more variable exploration, weaker feature binding, and greater flexibility under changing contingencies. From a unitary action-control perspective^40^, hippocampal neurogenesis may bias ideomotor behavior toward flexibility by reducing the dominance and competitive strength of strongly bound event files. By supporting representational differentiation and limiting the over-retrieval of prior action–effect associations, neurogenesis appears to modulate how event files compete for action control when contingencies change. This interpretation aligns with the concept of metacontrol^40–42^, which describes a higher-order regulatory dimension that biases action selection toward either persistence or flexibility within a single, unified action-control system. Within this framework, hippocampal neurogenesis can be understood as a biological factor that shifts the metacontrol state toward flexibility, attenuating the influence of strongly bound event files and promoting context-sensitive updating of action–effect representations. This aligns with computational^43,44^ and empirical accounts of adult-born dentate granule cells, which emphasize their roles in representational differentiation, interference resolution, and maintaining flexibility. Within the present framework, these functions can be understood as supporting the continual formation, refinement, and reconfiguration of event files. By facilitating the encoding of overlapping experiences as distinct representations, hippocampal neurogenesis may help preserve a balance between binding new action-effect relations and flexibly retrieving those that remain relevant. Importantly, the association between neurogenesis and both binding- and retrieval-related behavioral parameters suggests that hippocampal plasticity modulates the organization of action control in shaping future behavior. In doing so, adult hippocampal neurogenesis provides a neural mechanism through which ideomotor principles give rise to individual differences: animals with higher adult neurogenesis maintain more adaptable event-file repertoires, whereas animals with lower adult neurogenesis tend toward more rigid, strongly bound event-file repertoires. Adult hippocampal neurogenesis thus emerges as a key biological factor shaping how ideomotor learning unfolds, how behavioral individuality stabilizes, and how flexibility is preserved in dynamic environments.

One plausible mechanism linking the present histological and behavioral findings is the proposed contribution of adult hippocampal neurogenesis to pattern separation: the transformation of highly similar experiences into more distinct representations. Experimental suppression of adult neurogenesis impairs the discrimination of closely spaced locations and overlapping contexts, whereas increasing neurogenesis improves contextual discrimination^22,25,45^. This framework is particularly relevant to the IC paradigm, in which the spatial environment, sensory cues, and available responses remained largely unchanged across phases, while the effective action–outcome contingencies changed. More distinct representations of successive action–effect episodes could reduce interference from previously reinforced contingencies, support retrieval of the currently relevant relation, and thereby facilitate behavioral updating. Consistent with this interpretation, adult-born hippocampal neurons have also been implicated in the flexible use of spatial learning strategies and in changing previously learned responses when task demands change^46,47^. Although pattern separation was not measured directly in the present study, these findings provide a plausible mechanistic framework for the observed associations between hippocampal neurogenesis and flexible action control.

On a general level, the results support the view that ideomotor principles describe a species-general aspect of action organization. Basic exploration, learning, and adaptive behaviors in mice instantiate the same fundamental logic of event file binding and retrieval observed in humans. In this sense, behavioral individuality does not arise despite shared action-experience over time. The present findings position ideomotor theory as a unifying framework for understanding how action control, plasticity, and individuality co-emerge across levels of biological organization.

A few limitations should be considered. First, the sample size was modest, which may have reduced statistical power, particularly for smaller behavioral–histological associations. Nevertheless, the longitudinal design and repeated characterization of each animal provided a rich, multidimensional behavioral dataset that extends beyond conventional endpoint measures. Second, the two experimental cohorts differed in parts of their experimental histories, which may have contributed additional variability. However, the convergence of the main behavioral relationships across cohorts supports the presence of common underlying mechanisms rather than cohort-specific effects. Third, the associations between behavioral measures and adult hippocampal cell genesis are correlational and therefore do not establish the direction of causality. At the same time, previous experimental studies provide a strong mechanistic basis for this interpretation. Suppression or ablation of adult-born neurons impairs discrimination between similar spatial or contextual information and the flexible use of spatial learning strategies^25,45–47^. Conversely, experimentally increasing adult neurogenesis improves the discrimination of overlapping contexts and enhances pattern separation and cognitive flexibility^22,48^. These findings provide strong mechanistic support for the relationships observed here, although targeted suppression or enhancement of neurogenesis within the present behavioral paradigm will be required to test this link directly. Fourth, only female mice were examined, which limits generalization across sexes; however, their use enabled stable long-term group housing and minimally disturbed behavioral monitoring under socially consistent conditions. Finally, although the behavioral measures were interpreted within an ideomotor framework, the study was not designed to isolate every component of action–effect binding and retrieval experimentally. Rather, it provides a foundation for developing more targeted animal paradigms that directly manipulate these processes. These limitations define clear directions for future work but do not detract from the central observation that individual variation in hippocampal neurogenesis maps systematically onto distinct dimensions of adaptive action control.

In summary, the present work demonstrates that ideomotor principles of action control operate as organizing mechanisms of behavior in a naturalistic animal model and are systematically related to experience-dependent hippocampal plasticity. By examining longitudinal, self-generated behavior and cellular measures of adult hippocampal neurogenesis, we show how differences in exploration, learning flexibility, and behavioral individuality can be understood as consequences of how action–effect representations are formed, stabilized, and reused. These findings connect cognitive theory and neurobiology by identifying hippocampal neurogenesis as a key element in ideomotor principles. This positions ideomotor theory as a species-general framework that links action, plasticity, and individuality across levels of biological organization.

## Methods

### Animal husbandry

All animal housing and experimental procedures complied with relevant European and national legislation governing animal welfare (Tierschutzgesetz) and were approved by the responsible local authority (Landesdirektion Sachsen; file number TVV8/2019). Female C57BL/6JRj mice were obtained from Janvier Labs at five weeks of age. Upon arrival at the German Center for Neurodegenerative Diseases (DZNE) Dresden animal facility, mice were group-housed in standard cages (four animals per cage) for a two-week acclimation period. During the second week, animals were randomly assigned either to the IntelliCage (IC) condition, in which they received a subcutaneous injection of a glass-coated microtransponder (DataMars) in the neck region, or to continued housing in standard (STD) cages. Throughout the study, mice were maintained under a 12 h light/dark cycle with unrestricted access to food and water. Cage cleaning was performed weekly. Two separate experiments were conducted:

Experiment 1: Thirty-two female C57BL/6JRj mice, aged 5 weeks, were randomly assigned to either IC housing or STD housing for a duration of 11 weeks. To assess adult hippocampal neurogenesis, all mice received injections of the thymidine analog 5-chlorodeoxyuridine (CldU dissolved in 0.9% sodium chloride) three times at 9-hour time intervals, 4 weeks before the experiment ended. Following the experiment, IC mice were stratified by learning performance, with the two highest- and two lowest-performing learners selected for ex vivo hippocampal electrophysiology, along with two STD-housed mice. The remaining mice from both groups were perfused and processed for immunohistochemical quantification of adult hippocampal neurogenesis.

Experiment 2: Sixty-four female C57BL/6JRj mice, aged 5 weeks, were randomly assigned to either an IC (*n* = 32) or STD (*n* = 32) housing for a duration of 14 weeks. Two separate IC setups were used simultaneously, with the 32 IC-housed mice evenly distributed across them. Within each IC setup, mice were further divided into two experimental groups: a learning group (*n* = 24) and a learning-deprived group (*n* = 8). To quantify adult hippocampal neurogenesis, all mice received injections of IdU and CldU at 12.7 and 4 weeks, respectively, before the experiment ended. In the final two weeks, all mice underwent testing in the reference memory version of the Morris water maze to assess their spatial learning.

### IntelliCage (IC)

Mouse behavior was assessed using the IntelliCage system (TSE Systems, Bad Homburg, Germany), an automated, computer-controlled home-cage environment that enables continuous, long-term tracking of group-housed mice. The system accommodates up to 16 animals and contains four operant conditioning corners, each equipped with two drinking bottles containing sweetened water, two nose-poke sensors, and motorized doors that regulate access to the reward. Learning protocols were programmed via dedicated IntelliCage software, which was used to program adaptation and learning protocols where access to reward is based on a predefined combination of animal identity, visits to specific corners, and nose-poke patterns. Behavioral events, including corner visits, nose pokes, and licks, were automatically detected and time-stamped using RFID antennas, infrared nose-poke sensors, and lickometers, respectively. Each event was recorded together with the corresponding animal and corner identifiers. Two paper shelters and red plastic compartments were placed in the center of the cage. In addition to the sweetened water available in the corners during task phases, unsweetened water was available *ad libitum* from a bottle mounted in the upper grid of the cage near the *ad libitum* food. The following task modules were implemented sequentially using the IntelliCage software:

1. Free adaptation: All doors remained open, allowing unrestricted access to sweetened water in all four corners.
2. Nosepoke adaptation: Access to the reward required a nose poke, which opened the door for 5 s. A corner had to be exited before the sensor could be reactivated.
3. Drinking time adaptation: Reward access was limited to a 4.5 h window during the dark phase (22:30–03:00 for IC1; 21:00–03:00 for IC2).
4. Place learning with 3 corners correct (PL3CC): Rewards were available in three corners following a nose poke.
5. Place learning with 1 corner correct (PL1CC): Only a single corner was rewarded after a nose poke; the previously incorrect corner became the only correct corner.
6. Patrolling with nosepoke: The position of the rewarded correct corner shifted in either a clockwise or anticlockwise manner.
7. Reverse patrolling with nosepoke: The direction of the rewarded-corner sequence was reversed relative to the previous patrolling task.
8. Serial Reversal Learning Task: Two diagonally opposite corners were rewarded, requiring animals to shuttle between them. The assignment of diagonally opposite correct and incorrect corners was reversed every 4 days.
9. Free days: No learning contingencies were imposed, allowing recovery between task phases.
10. Competition Task: All animals have restricted water access to a single designated corner for one hour per day. During this limited access period, they compete for drinking opportunities, leading to the emergence of hierarchical structures. The order in which animals gain access to water at the start of each session reveals their relative ranks within the group.

### Tissue preparation and immunohistochemistry

Animals were deeply anesthetized using ketamine (WDT; 100 mg/kg) and xylazine (Serumwerk Bernburg AG; 10 mg/kg) and subsequently perfused transcardially with 0.9% saline followed by 4% paraformaldehyde (PFA) in phosphate buffer (pH 7.4). Brains were extracted, post-fixed overnight in PFA at 4 °C, and transferred in 30% sucrose for a minimum of two days. Coronal sections (40 µm) were cut on a sliding microtome equipped with a dry ice–cooled stage (Leica SM2000R) and stored at 4 °C in cryoprotectant solution containing 25% ethylene glycol and 25% glycerol in 0.1 M phosphate buffer (pH 7.4).

CldU-positive cells were visualized using a peroxidase-based immunohistochemical approach on free-floating sections. Endogenous peroxidase activity was quenched by incubation in 0.6% hydrogen peroxide for 30 min. For antigen retrieval, DNA denaturation was achieved by treating sections with pre-warmed 2.5 M hydrochloric acid for 30 min at 37 °C, followed by extensive washing steps. Non-specific binding was blocked by incubating sections in tris-buffered saline (TBS) supplemented with 10% donkey serum (Jackson ImmunoResearch Labs) and 0.2% Triton X-100 (Carl Roth) for 1 h at room temperature. Sections were then incubated overnight at 4 °C with a monoclonal rat anti-CldU antibody (1:500: BD Biosciences), followed by biotinylated secondary antibodies for (Jackson ImmunoResearch Labs) 2 h at room temperature. Antibodies were diluted in TBS containing 3% donkey serum and 0.2% Triton X-100. Signal amplification and visualization were performed using the VECTASTAIN Elite ABC kit (9 µg/ml of each component: Vector Laboratories, LINARIS) and diaminobenzidine (0.075 mg/ml; Sigma-Aldrich) with 0.04% nickel chloride as chromogen. All rinses were carried out in TBS. Finally, sections were mounted on glass slides, cleared with Neo-Clear (Millipore), and cover slipped with Neo-Mount (Millipore). Quantification of CldU-labeled cells was performed on every sixth section throughout the rostrocaudal extent of the dentate gyrus (DG) using bright-field microscopy (Leica DM 750).

### IC behavioral analysis

The IC software outputs a raw data file with descriptive information about each animal’s visit to the corners, which allows the computation of the variables per day listed below.

#### Roaming entropy

In the adaptation phase, exploratory activity was examined by calculating roaming entropy. Corner visit frequencies were converted to probabilities ${p}_{i,j,t}$ of a mouse *i* being found at a corner *j* on a day *t*. Shannon entropy of the roaming distribution was calculated as roaming entropy

$${{{\mathrm{RE}}}}_{(i,t)}=-{\sum}_{(j=1)}^{4}\frac{{p}_{i,j,t}{{{\mathrm{log}}}}({p}_{i,j,t})}{{{\mathrm{log}}}(4)}$$

Dividing the entropy by log(4) which is the number of corners in the IC scales the RE to the range between zero and one.

#### Corner preference

For each mouse, the total number of visits to each corner was counted and converted into proportions by normalizing visit counts to the total number of visits made on that day. The corner with the highest visit proportion was defined as the preferred corner for that day. In cases where multiple corners had equal proportions, ties were excluded.

#### Corner-to-corner transition probability matrix

For each animal, a corner-to-corner transition matrix was constructed based on the sequence of visits during the analysis period. The matrix captured the frequency with which each corner (1–4) was visited immediately following departure from every other corner. Transition frequencies were normalized to account for overall visit distribution, allowing comparison across animals with different activity levels.

#### Repetition bias

Repetition bias was derived from the diagonal elements of the corner transition matrix, which represent instances in which an animal re-entered the same corner it had recently left. Observed re-entry frequencies were compared to expected frequencies calculated from the marginal visit probabilities for each corner^35^.

$${Repetition}\,{bias}={log}\frac{{sum}\,{of}\,{observed}\,{re}-{entries}}{{sum}\,{of}\,{expected}\,{re}-{entries}}$$

Expected values reflect the likelihood of re-entry under random corner selection (e.g., 25% if all corners are visited equally often). Repetition bias yields a measure centered around zero. Positive values indicate a higher-than-expected tendency to repeatedly visit the same corner, and vice versa.

#### Regularity index

The regularity index was computed from the off-diagonal elements of the corner transition matrix, which describe transitions between different corners. For each non-reentry transition, deviations between observed and expected transition frequencies were calculated and squared, then normalized by the expected frequency. These values were summed across all off-diagonal transitions and divided by the total number of non-reentry transitions to obtain a single regularity measure per animal^35^.

$${Regularity}\,{index}=\sqrt{\frac{1}{{N}_{{ns}}}{\sum}_{i\ne j}\frac{{O}_{{ij}}-{E}_{{ij}}}{{E}_{{ij}}}}$$

where ${O}_{{ij}}$ and ${E}_{{ij}}$ denote the observed and expected transition frequencies from corner i to corner j, respectively, and ${N}_{{ns}}$ is the total number of non–same-corner transitions (i≠j). This index increases when visit sequences follow structured or predictable patterns (e.g., ordered visits of corners) and is low when transitions are uniformly distributed across all possible corner pairs.

#### Learning

In the learning phase, the percentage of correct visits

$$\% {correct}\,{{\mathrm{visits}}}=\frac{{{\mathrm{correct\; visits}}}}{{{\mathrm{total\; visits}}}}\times 100$$

was used as a metric to analyze the learning behavior of individual mice during all tasks. A correct visit is defined as a visit to a correct corner followed by more than one nosepoke, which indicates a reward-seeking behavior as opposed to just exploratory behavior.

#### Persistence

To assess the persistent behavior of individual mice in visiting the previous learning rule when a new task starts, the percentage of times mice visited the previously assigned correct corner was calculated:

$$\% {visits}\,{to}\,{previous}\,{correct}\,{corners}=\frac{{{{\rm{visits}}}}\; {{{\rm{to}}}}\; {{{\rm{correct}}}}\; {{{\rm{corners}}}}\; {{{\rm{of}}}}\; {{{\rm{previous}}}}\; {{{\rm{task}}}}}{{{{\rm{total}}}}\; {{{\rm{visits}}}}}\times 100$$

#### Flexibility Index

To quantify the tradeoff between and learning and persistence, flexibility index per animal was calculated as the difference between learning score and persistence score across tasks

$${Flexibility}\,{Index}\,\left({FI}\right)= \% {correct}\,{visits}- \% {visits}\,{to}\,{previous}\,{correct}\,{corners}$$

### Principal component analysis

To reduce the dimensionality of behavioral measures and identify shared structure across individuals, principal component analysis (PCA) was performed on animal-level summary metrics. Prior to PCA, animals with missing or non-finite values in any numeric variable were excluded. All variables were z-standardized before analysis to ensure equal weighting. PCA was computed using singular value decomposition (prcomp function in R), excluding animal identifiers from the input matrix. The proportion of variance explained by each principal component was extracted from the eigenvalues, and scree plots were used to visualize variance distribution across components. Variable loadings were examined to assess the contribution of each behavioral measure to the principal components. Loading plots and correlation circles were generated to visualize variable contributions in PC space, with arrow lengths reflecting the strength of association between original variables and the principal components. Individual PCA scores (coordinates of each animal in PC space) were extracted and used for visualization and subsequent statistical analyses. For descriptive classification, animals were split into high and low binding/retrieval groups based on a median split of PC1 scores. PCA scores were further related to behavioral and neurobiological measures using Pearson correlation analyses.

### Statistical analysis

Statistical analyses were conducted using the R software (R Core Team, 2014). To compare longitudinal data means, a rank-based nonparametric test was employed utilizing the “nparLD” function from the “nparLD” package^49^. This test provides analysis of variance (ANOVA)-type statistics for time, group, and their interaction. Post hoc pairwise comparisons were executed using Wilcoxon’s test. Variances between groups were compared using the Brown-Forsythe test, conducted with the “leveneTest” function from the “car” package. All statistical tests were two-tailed, and significance was considered at *P* < 0.05. The data visualizations were generated using the “ggplot2” package^50^. In the box-whisker plots, the centerline represents the median. The upper and lower hinges indicate the first and third quartiles, respectively. The upper whisker extends from the hinge to the largest value within 1.5 times the interquartile range (IQR), while the lower whisker extends from the hinge to the smallest value within 1.5 times the IQR.

### Reporting summary

Further information on research design is available in the Nature Portfolio Reporting Summary linked to this article.

## Supplementary information

Supplemental Material

reporting summary

## Data Availability

Behavioral data from IntelliCage experiment 1 have been deposited in the DRYAD repository and are accessible through the identifiers: https://doi.org/10.5061/DRYAD.XGXD254RB; https://doi.org/10.5061/dryad.xgxd254rb. Behavioral data from IntelliCage experiment 2 have been deposited in the OSF repository^51^ and are accessible via the following link: https://doi.org/10.17605/OSF.IO/4RCKA.

## Code availability

The code used for data processing, statistical analyses, and figure generation has been deposited in the OSF repository^51^ and is accessible via the following link: https://doi.org/10.17605/OSF.IO/4RCKA.
