# Supplementary material for: Adult hippocampal neurogenesis shapes ideomotor action control in mice
Source: Commun Biol. 2026 Oct 7;9:1307. doi: 10.1038/s42003-026-11112-z (PMC13645787; doi:10.1038/s42003-026-11112-z)
Supplement: Supplementary file 1 [file 42003_2026_11112_MOESM1_ESM.pdf]

Supplementary information for

Adult hippocampal neurogenesis shapes ideomotor action control in mice

Warsha Barde[1,2,3,4], Gerd Kempermann[1,2], Christian Beste[3,4]

Corresponding author: christian.beste@ukdd.de (C.B.)

The PDF file includes:

Figs. S1 to S4

Tables S1 to S6

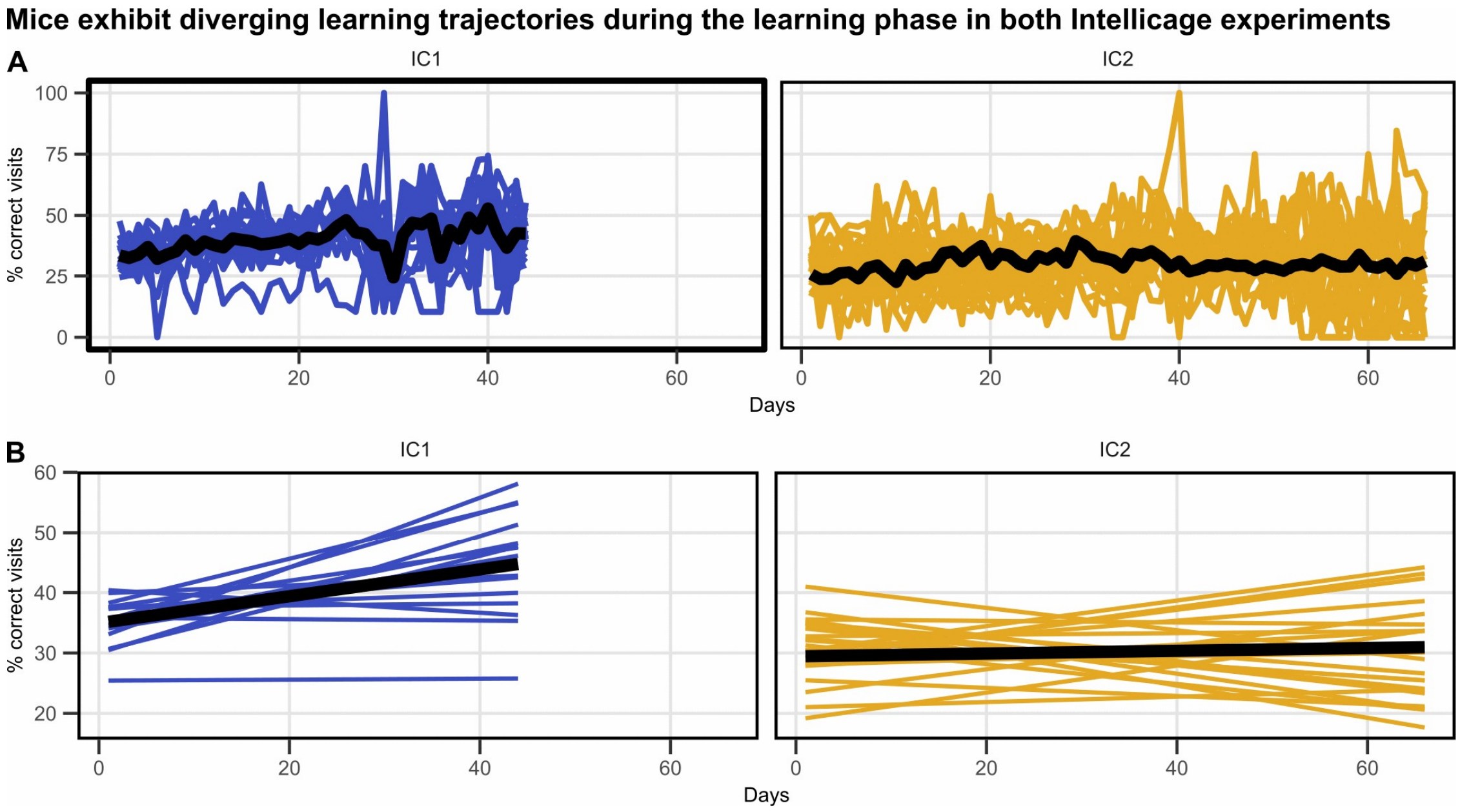

Supplementary Figure 1. Diverging learning trajectories across the entire learning phase.

(A) Raw Individual learning trajectories shown as % correct visits per day, centered on chance level for each task. **(B)** Learning trajectories with linear fits for each animal. Solid black line indicates the group mean trajectory.

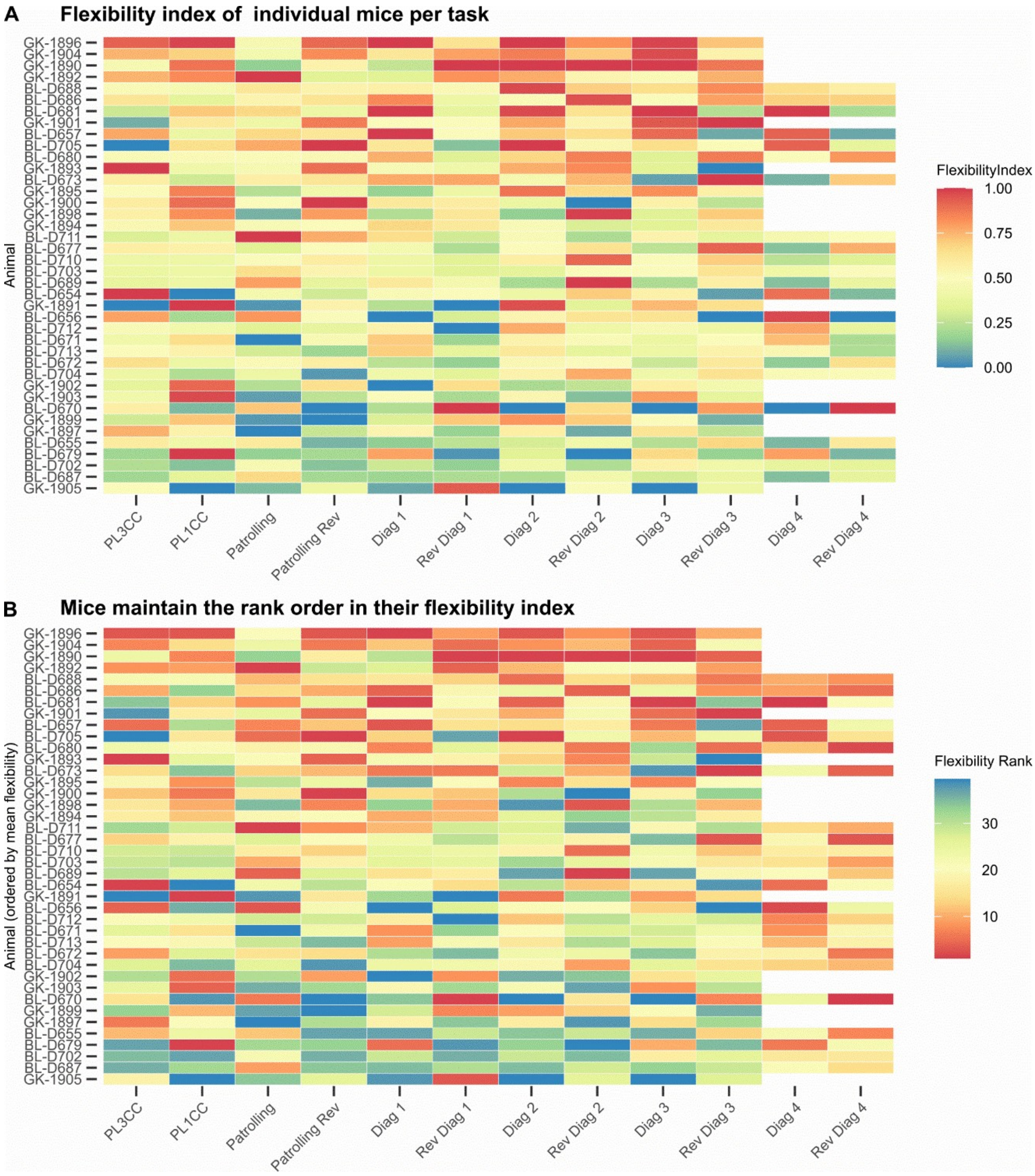

Supplementary Figure 2. Stable inter-individual differences in the flexibility index of mice.

(A) Flexibility index of individual mice calculated as the difference between learning score and persistence score across tasks, captures each animal's behavioral adaptability. **(B)** Rank order of mice based on their flexibility index for each task exhibits remarkable stability: animals with high flexibility consistently maintain a high rank, whereas animals with lower flexibility retain lower ranks, indicating stable individual differences in adaptive behavior.

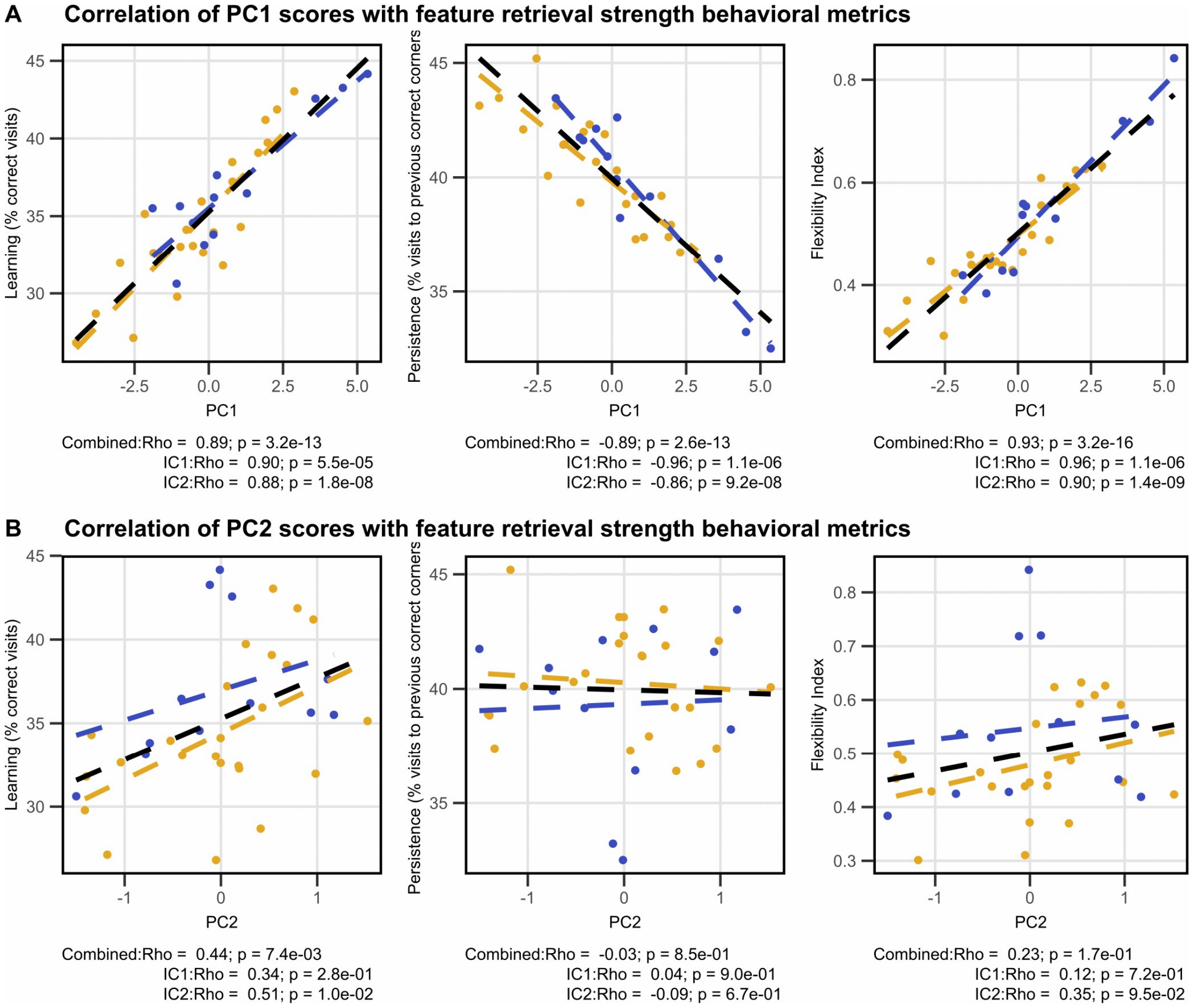

Supplementary Figure 3. Correlations between PCA axes and behavioral measures of feature retrieval strength.

(A) PC1 scores are strongly positively correlated with learning and flexibility measures, and negatively correlated with persistence metrics. **(B)** PC2 scores show a strong positive correlation with learning success, but weaker and non-significant correlation with persistence and flexibility.

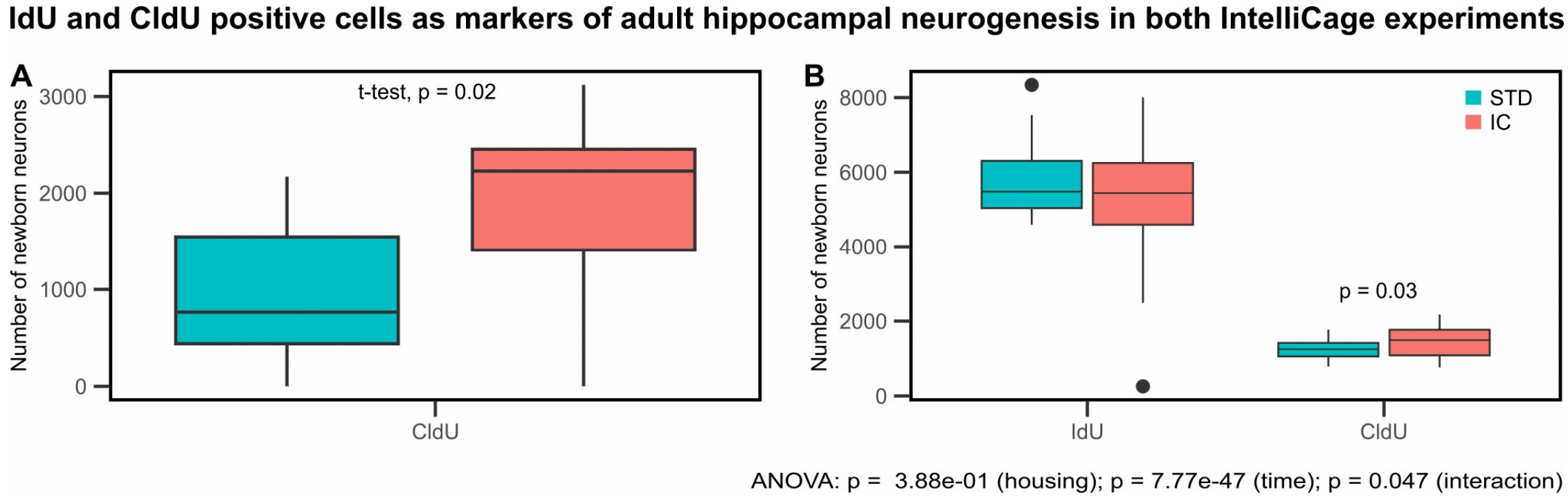

Supplementary Figure 4. Adult hippocampal neurogenesis.

(A) IC mice showed higher mean and variance in the number of new hippocampal neurons (CldU-positive) compared to STD mice (STD, n = 14; IC, n = 12). Box and whisker plots: center line, median; upper and lower hinges, first and third quartiles; whiskers, highest and lowest values within 1.5 times the interquartile range (IQR) outside hinges. **(B)** Quantification of IdU-positive cells in the DG at the early phase (7.7 weeks) shows no significant differences between IC and STD mice, indicating comparable early neurogenesis rates. At the late phase (16 weeks), IC mice exhibit a significantly higher number of CldU-positive cells compared to STD mice.

Supplementary Table 1. PCA loadings for composite feature binding strength.

	PC1	PC2	PC3	PC4	PC5
**RE**	0.646356	-0.03601	0.040016	-0.06517	-0.75834
**RE slope**	0.323553	0.578598	0.273483	0.66597	0.2055
**Total Corner Switches**	0.245534	-0.61793	-0.46753	0.558348	0.165964
**Repetitive Bias**	0.441758	0.37433	-0.61213	-0.3998	0.360806
**Regularity Index**	-0.47128	0.376793	-0.57473	0.283977	-0.47431

Loadings of roaming entropy (RE), RE slope, total corner switches, repetitive bias and regularity index on the five principal components.

Supplementary Table 2. Task-wise correlations between persistence and learning (Pearson r, p-values).

Task	r	p
**PL1CC**	−1.00	$< 1 \times 10^{-12}$
**PL3CC**	−1.00	$< 1 \times 10^{-12}$
**Patrolling**	−0.09	6.06×10^{-1}
**Patrolling Rev**	−0.25	1.27×10^{-1}
**Diag 1**	−0.19	2.43×10^{-1}
**Rev Diag 1**	−0.69	1.42×10^{-6}
**Diag 2**	−0.85	9.22×10^{-12}
**Rev Diag 2**	−0.51	9.42×10^{-4}
**Diag 3**	−0.73	1.47×10^{-7}
**Rev Diag 3**	−0.71	4.92×10^{-7}
**Diag 4**	−0.77	1.07×10^{-5}
**Rev Diag 4**	−0.92	1.89×10^{-10}

Pearson correlation coefficients (r) and corresponding p values are reported for each IC learning task

Supplementary Table 3. PCA loadings for composite feature retrieval strength.

	**PC1**	**PC2**	**PC3**
**Flexibility Index**	0.61873	-0.00073	0.785604
**Learning score (% correct visits)**	0.555608	-0.70657	-0.43825
**Persistence score (% visits to previous correct corners)**	-0.5554	-0.70764	0.436768

Loadings of the flexibility index, learning score and persistence score on the three principal components.

Supplementary Table 4. Correlations between hippocampal neurogenesis and behavioral measures.

**Behavioral metric**	**All Mice**	**IC1**	**IC2**
Mean RE	r = 0.32, p = 0.054	r = 0.27, p = 0.40	r = 0.35, p = 0.093
RE slope	r = 0.34, p = 0.041	r = 0.15, p = 0.65	r = 0.43, p = 0.037
Preferred corner switching	r = 0.36, p = 0.033	r = 0.48, p = 0.11	r = 0.31, p = 0.14
Repetition bias	r = 0.11, p = 0.54	r = 0.44, p = 0.15	r = −0.02, p = 0.92
Regularity index	r = −0.12, p = 0.50	r = −0.29, p = 0.36	r = −0.04, p = 0.85
Learning score	r = 0.50, p = 0.002	r = 0.45, p = 0.14	r = 0.55, p = 0.0057
Persistence	r = −0.23, p = 0.18	r = −0.26, p = 0.42	r = −0.22, p = 0.30
Flexibility index	r = 0.38, p = 0.024	r = 0.40, p = 0.20	r = 0.40, p = 0.052
Feature binding strength	r = 0.30, p = 0.047	r = 0.44, p = 0.15	r = 0.26, p = 0.14
Feature retrieval strength	r = 0.34, p = 0.044	r = 0.30, p = 0.34	r = 0.38, p = 0.064

Pearson correlation coefficients (r) and corresponding p values are reported for the full sample and separately for the IC1 and IC2 cohorts.

Supplementary Table 5. Ranks of individual animals across feature binding behavioral metrics.

**Animal**	**Neurogenesis (CldU)**	**RE**	**RE slope**	**Preferred corner switch**	**Repetition bias**	**Regularity index**	**Binding PC1 score**
GK-1895	4	19	21	16	20	31	18
GK-1896	26	3	9	20	7	35	4
GK-1897	22	29	28	29	8	22	28
GK-1898	12	2	27	21	4	26	7
GK-1899	25	9	29	6	19	21	16
GK-1900	30	33	32	22	32	12	40
GK-1902	32	35	16	23	35	2	41
GK-1903	***33***	***22***	***14***	***33***	***17***	***13***	***26***
GK-1904	***1***	***15***	***12***	***17***	***11***	***7***	***19***
GK-1890	16	16	34	27	6	25	21
GK-1891	20	26	31	19	23	14	31
GK-1893	9	17	13	28	14	36	15
BL-D670	10	31	24	8	9	6	25
BL-D672	29	7	20	1	24	24	12
BL-D673	***2***	***13***	***5***	***24***	***30***	***19***	***22***
BL-D671	35	25	10	31	18	33	27

BL-D679	18	14	23	25	2	23	13
BL-D681	11	4	4	9	3	20	5
BL-D677	19	6	2	3	10	32	3
BL-D680	8	12	11	5	25	29	11
BL-D655	14	27	19	7	27	16	29
BL-D657	24	20	8	30	28	34	24
BL-D654	31	28	17	34	15	4	32
BL-D656	23	23	6	36	31	5	34
BL-D713	7	8	1	15	16	11	6
BL-D712	6	5	15	14	5	30	9
BL-D711	28	18	33	13	29	3	33
BL-D710	5	1	3	12	1	27	1
BL-D703	21	10	30	32	21	18	23
BL-D704	***36***	***34***	***26***	***26***	***22***	***15***	***36***
BL-D705	13	36	18	35	34	1	43
BL-D702	34	21	36	11	33	17	39
BL-D688	3	32	22	4	36	9	42
BL-D687	15	24	35	2	12	8	30
BL-D686	17	30	7	18	26	28	20
BL-D689	27	11	25	10	13	10	17

Individual animals are ranked according to adult hippocampal neurogenesis, measured by CldU-labelled cell counts, and each constituent behavioral metric: RE, RE slope, preferred-corner switching, repetition bias and regularity index, together with the composite feature-binding PC1 score.

Supplementary Table 6. Ranks of individual animals across feature retrieval behavioral metrics

Animal	Neurogenesis (CldU)	Learning score	Persistence score	Flexibility Index	Retrieval PC1 score
GK-1895	4	10	27	12	14
GK-1896	26	1	36	1	1
GK-1897	22	32	12	32	27
GK-1898	12	12	24	14	9
GK-1899	25	16	3	31	31
GK-1900	30	22	21	13	17
GK-1902	32	18	8	28	22
GK-1903	***33***	***23***	***16***	***29***	***18***
GK-1904	***1***	***4***	***33***	***2***	***3***
GK-1890	16	2	35	3	2
GK-1891	20	15	13	21	25
GK-1893	9	13	6	10	15
BL-D670	10	17	20	30	32
BL-D672	29	24	17	26	21
BL-D673	***2***	***11***	***31***	***11***	***11***
BL-D671	35	28	14	24	28
BL-D679	18	34	2	34	35
BL-D681	11	7	28	6	6
BL-D677	19	19	30	16	10
BL-D680	8	6	29	9	7
BL-D655	14	27	5	33	30
BL-D657	24	9	23	7	12
BL-D654	31	33	25	20	26
BL-D656	23	30	9	22	34
BL-D713	7	25	10	25	24
BL-D712	6	20	7	23	23
BL-D711	28	31	26	15	13
BL-D710	5	14	11	17	20
BL-D703	21	21	18	18	16
BL-D704	***36***	***26***	***19***	***27***	***19***
BL-D705	13	8	22	8	8
BL-D702	34	36	4	35	36
BL-D688	3	3	34	4	4
BL-D687	15	35	1	36	33

BL-D686	17	5	32	5	5
BL-D689	27	29	15	19	29

Individual animals are ranked according to adult hippocampal neurogenesis, measured by CldU-labelled cell counts, and each constituent behavioral metric: learning score, persistence score and flexibility index, together with the composite feature-retrieval PC1 score.
