# Supplementary material for: Adult hippocampal neurogenesis shapes ideomotor action control in mice
Source: Commun Biol. 2026 Oct 7;9:1307. doi: 10.1038/s42003-026-11112-z (PMC13645787; doi:10.1038/s42003-026-11112-z)
Supplement: Supplementary file 2 [file 42003_2026_11112_MOESM2_ESM.pdf]

nature portfolio

Corresponding author(s): Christian Beste

Last updated by author(s): May 18, 2026

Reporting Summary

Nature Portfolio wishes to improve the reproducibility of the work that we publish. This form provides structure for consistency and transparency in reporting. For further information on Nature Portfolio policies, see our Editorial Policies and the Editorial Policy Checklist.

Statistics

For all statistical analyses, confirm that the following items are present in the figure legend, table legend, main text, or Methods section.

n/a	Confirmed	
☐	☒	The exact sample size (*n*) for each experimental group/condition, given as a discrete number and unit of measurement
☐	☒	A statement on whether measurements were taken from distinct samples or whether the same sample was measured repeatedly
☐	☒	The statistical test(s) used AND whether they are one- or two-sided<br>*Only common tests should be described solely by name; describe more complex techniques in the Methods section.*
☐	☒	A description of all covariates tested
☐	☒	A description of any assumptions or corrections, such as tests of normality and adjustment for multiple comparisons
☐	☒	A full description of the statistical parameters including central tendency (e.g. means) or other basic estimates (e.g. regression coefficient) AND variation (e.g. standard deviation) or associated estimates of uncertainty (e.g. confidence intervals)
☐	☒	For null hypothesis testing, the test statistic (e.g. *F*, *t*, *r*) with confidence intervals, effect sizes, degrees of freedom and *P* value noted<br>*Give P values as exact values whenever suitable.*
☒	☐	For Bayesian analysis, information on the choice of priors and Markov chain Monte Carlo settings
☒	☐	For hierarchical and complex designs, identification of the appropriate level for tests and full reporting of outcomes
☐	☒	Estimates of effect sizes (e.g. Cohen's *d*, Pearson's *r*), indicating how they were calculated

Our web collection on statistics for biologists contains articles on many of the points above.

Software and code

Policy information about availability of computer code

Data collection	Behavioral data were collected using the IntelliCage system (TSE Systems), which automatically records RFID-based corner visits, nose pokes and licking behavior in group-housed mice.
Data analysis	Data processing, statistical analyses and figure generation were performed in R using custom scripts. The code used for data processing, statistical analyses and figure generation has been deposited in the OSF repository and is accessible via the following link: https://osf.io/4rcka/overview?view_only=627a72769a6544f0af0e1a84e3e7ec22. The repository is currently available as a private view-only link for review and will be made public upon publication.

For manuscripts utilizing custom algorithms or software that are central to the research but not yet described in published literature, software must be made available to editors and reviewers. We strongly encourage code deposition in a community repository (e.g. GitHub). See the Nature Portfolio guidelines for submitting code & software for further information.

Data

Policy information about availability of data

All manuscripts must include a data availability statement. This statement should provide the following information, where applicable:

- Accession codes, unique identifiers, or web links for publicly available datasets
- A description of any restrictions on data availability
- For clinical datasets or third party data, please ensure that the statement adheres to our policy

Behavioral data from IntelliCage experiment 1 have been deposited in the DRYAD repository and are accessible through the identifiers: doi:10.5061/DRYAD.XGXD254RB; doi:10.5061/dryad.xgxd254rb. Behavioral data from IntelliCage experiment 2 have been deposited in the OSF repository and are accessible via the following link: https://osf.io/4rcka/overview?view_only=627a72769a6544f0af0e1a84e3e7ec22.

Research involving human participants, their data, or biological material

Policy information about studies with human participants or human data. See also policy information about sex, gender (identity/presentation), and sexual orientation and race, ethnicity and racism.

Reporting on sex and gender	This study did not involve human participants.
Reporting on race, ethnicity, or other socially relevant groupings	The study did not involve human participants.
Population characteristics	The study did not involve human participants.
Recruitment	The study did not involve human participants.
Ethics oversight	The study did not involve human participants.

Note that full information on the approval of the study protocol must also be provided in the manuscript.

Field-specific reporting

Please select the one below that is the best fit for your research. If you are not sure, read the appropriate sections before making your selection.

☒ Life sciences ☐ Behavioural & social sciences ☐ Ecological, evolutionary & environmental sciences

For a reference copy of the document with all sections, see nature.com/documents/nr-reporting-summary-flat.pdf

Life sciences study design

All studies must disclose on these points even when the disclosure is negative.

Sample size	No statistical method was used to predetermine sample size. Sample sizes were guided by previous IntelliCage studies and by the recommended maximum group size for IntelliCage housing, which is 16 mice per IntelliCage to ensure sufficient enrichment space. The study comprised two independent IntelliCage experiments with matched standard-housed control groups. Experiment 1 included n = 16 IntelliCage mice and n = 16 standard-housed controls. Experiment 2 included n = 32 IntelliCage mice, housed across two simultaneously operated IntelliCage setups, and n = 32 standard-housed controls.
Data exclusions	In experiment 2, animals that did not undergo the learning task sequence were excluded from learning-related behavioural analyses. Behavioural records affected by technical errors, incomplete RFID detection or invalid timestamps were excluded during preprocessing.
Replication	Behavioural analyses were performed across two independent IntelliCage experiments. The experiments differed in some procedural details, including duration, task sequence and thymidine-analogue labelling protocol, providing an opportunity to assess whether the behavioural-neurogenesis relationships were consistent across independent cohorts. Where applicable analyses were reported for individual experiments and for pooled data.
Randomization	Before the start of the experiment, animals were randomly assigned to housing conditions - IntelliCage or standard housing. In experiment 2, IntelliCage-housed animals were distributed across two simultaneously operated IntelliCage setups.
Blinding	Blinding was not applicable in this study because all behavioral data were collected using fully automated recording systems without experimenter intervention during data acquisition. Behavioral measures were computed from time-stamped, sensor-based data using identical analysis pipelines. As a result, there was no subjective assessment or manual scoring and blinding was therefore not relevant.

Reporting for specific materials, systems and methods

We require information from authors about some types of materials, experimental systems and methods used in many studies. Here, indicate whether each material, system or method listed is relevant to your study. If you are not sure if a list item applies to your research, read the appropriate section before selecting a response.

Materials & experimental systems

n/a	Involved in the study
☒	☐ Antibodies
☒	☐ Eukaryotic cell lines
☒	☐ Palaeontology and archaeology
☐	☒ Animals and other organisms
☒	☐ Clinical data
☒	☐ Dual use research of concern
☒	☐ Plants

Methods

n/a	Involved in the study
☒	☐ ChIP-seq
☒	☐ Flow cytometry
☒	☐ MRI-based neuroimaging

Animals and other research organisms

Policy information about studies involving animals; ARRIVE guidelines recommended for reporting animal research, and Sex and Gender in Research

Laboratory animals	Across both IntelliCage experiments, behaviour was assessed in C57BL/6JRj mice obtained at 4 weeks of age.
Wild animals	The study did not involve wild animals.
Reporting on sex	The study was conducted in female mice. Females were selected because the IntelliCage paradigm requires stable long-term group housing, whereas group housing of male mice can increase the risk of inter-individual aggression, stress and injury, which could compromise animal welfare and confound behavioural measurements.
Field-collected samples	The study did not involve samples collected from the field.
Ethics oversight	All animal husbandry and experimental procedures were conducted in accordance with applicable European and national animal welfare regulations (Tierschutzgesetz). The study was approved by the local authority, Landesdirektion Sachsen, under file number TVV8/2019.

Note that full information on the approval of the study protocol must also be provided in the manuscript.

Plants

Seed stocks	The study did not involve plant specimens.
Novel plant genotypes	The study did not involve plant specimens.
Authentication	The study did not involve plant specimens.
